# *Pseudomonas fluorescens* Showing Antifungal Activity against *Macrophomina phaseolina,* a Severe Pathogenic Fungus of Soybean, Produces Phenazine as the Main Active Metabolite

**DOI:** 10.3390/biom11111728

**Published:** 2021-11-19

**Authors:** Stefany Castaldi, Marco Masi, Francisco Sautua, Alessio Cimmino, Rachele Isticato, Marcelo Carmona, Angela Tuzi, Antonio Evidente

**Affiliations:** 1 Dipartimento di Biologia, Università di Napoli Federico II, Complesso Universitario Monte S. Angelo, Via Cintia 4, 80126 Napoli, Italy; isticato@unina.it; 2 Dipartimento di Scienze Chimiche, Università di Napoli Federico II, Complesso Universitario Monte S. Angelo, Via Cintia 4, 80126 Napoli, Italy; marco.masi@unina.it (M.M.); alessio.cimmino@unina.it (A.C.); angela.tuzi@unina.it (A.T.); evidente@unina.it (A.E.); 3 Cátedra de Fitopatología, Facultad de Agronomía, Universidad de Buenos Aires, Ciudad Autónoma de Buenos Aires C1417DSE, Argentina; sautua@agro.uba.ar (F.S.); carmonam@agro.uba.ar (M.C.)

**Keywords:** *Pseudomonas fluorescens*, *Macrophomina phaseolina*, phenazine, phenazine analogs and derivatives, soybean pathogens, antifungal activity, SAR

## Abstract

*Pseudomonas fluorescens* 9 and *Bacillus subtilis* 54, proposed as biofungicides to control *Macrophomina phaseolina*, a dangerous pathogen of soybean and other crops, were grown in vitro to evaluate their ability to produce metabolites with antifungal activity. The aim of the manuscript was to identify the natural compounds responsible for their antifungal activity. Only the culture filtrates of *P. fluorescens* 9 showed strong antifungal activity against *M. phaseolina*. Its organic extract contained phenazine and mesaconic acid (**1** and **2**), whose antifungal activity was tested against *M. phaseolina*, as well as *Cercospora nicotianae* and *Colletotrichum truncatum*, other pathogens of soybean; however, only compound **1** exhibited activity. The antifungal activity of compound **1** was compared to phenazine-1-carboxylic acid (PCA, **3**), 2-hydroxyphenazine (2-OH P, **4**), and various semisynthetic phenazine nitro derivatives in order to perform a structure–activity relationship (SAR) study. PCA and phenazine exhibited the same percentage of growth inhibition in *M. phaseolina* and *C. truncatum*, whereas PCA (**3**) showed lower activity against *C. nicotianae* than phenazine. 2-Hydroxyphenazine (**4**) showed no antifungal activity against *M. phaseolina*. The results of the SAR study showed that electron attractor (COOH and NO_2_) or repulsor (OH) groups significantly affect the antifungal growth, as well as their α- or β-location on the phenazine ring. Both PCA and phenazine could be proposed as biopesticides to control the soybean pathogens *M. phaseolina*, *C. nicotianae*, and *C. truncatum*, and these results should prompt an investigation of their large-scale production and their suitable formulation for greenhouse and field applications.

## 1. Introduction

Food demand has increased with the gradual growth of the world population, which is expected to reach almost 10 billion by 2050 [1,2]. Likewise, agricultural production has increased over time with the development of technology and biotechnology innovations. As a consequence, the environmental pollution of soil and water has negatively and significantly affected the quality and quantity of agricultural production [3,4]. Microbial pathogens, weeds, and animal pests among the biotic stresses are responsible for heavy losses affecting crop yields. The use of chemical pesticides (herbicides, insecticides, fungicides, bactericides, etc.) has increased over time. Their massive use during the last 5–6 decades has become a threat to environmental preservation and a severe risk to human and animal health. Among the biotic stresses, fungal pathogens are the main causal agents of crop diseases. Their ability to produce phytotoxins plays an important role in inducing plant disease symptoms [5,6].

*Macrophomina phaseolina* (Tassi) Goid is one of the most virulent and yield-limiting phytopathogens [7,8]. This necrotrophic fungus can infect more than 500 plant species in more than 100 families causing dry root and stem rot, known as charcoal rot (CR) [9]. CR is an important disease of leguminous crops such as soybean, chickpea, peanut, alfalfa, bean, and pea, as well as of cereals such as maize, sorghum, and sugarcane. Recently, *M. phaseolina* was reported as responsible for grapevine decline in Iran [10]. The first studies on the phytotoxins produced by *M. phaseolina* reported the purification and the properties of the exotoxin produced by the pathogen isolated in India [11]. Subsequently, the main phytotoxin structure, an eremophilane sesquiterpenoid named phaseolinone, was determined [12]. Recently, from strain 2013-1 of *M. phaseolina*, obtained from infected soybean roots growing in Pergamino, Argentina, two new phytotoxic penta- and tetra-substituted cyclopentenones, named phaseocyclopentenones A and B, together with guignardone A, were isolated. [13].

Fungi belonging to other genera such as *Cercospora* and *Colletotrichum* are causal agents of several diseases of soybean in Argentina and Brazil, which have also developed resistance or changes in sensitivity to commonly used fungicides [14,15,16,17,18].

A collection of bacteria were isolated to soybean plants and screened for their antagonistic activity against *M. phaseolina* aiming to avoid the use of chemical fungicides. Two of them, identified as *Pseudomonas fluorescens* 9 and *Bacillus subtilis* 54 resulted most promising and were tested further using in vitro assays, as well as in the greenhouse. In particular, *P. fluorescens* 9 showed a greater reduction in disease than *B. subtilis* [19]. These results are not surprising, as the microbial antagonisms performed by beneficial bacteria and fungi against different phytopathogens are well known [20]. In fact, some bacterial metabolites have shown antifungal activity against pathogens of some important crops, such as phenazine-1-carboxylic acid and 2-hydroxyphenazine, which are produced by *Pseudomonas chlororaphis* subsp. *aureofaciens* strain M7. These metabolites, compared to some semisynthetic phenazine-1-carboxylic acid (PCA) derivatives, were assayed against a group of crop and forest plant-pathogenic fungi. Among the compounds tested, PCA was active against almost all tested pathogens. Instead, 2-hydroxyphenazine (2-OH P) weakly inhibited a few fungal species. The results of a structure–activity relationship (SAR) study, testing the four semisynthetic derivatives of PCA, showed that the carboxyl group is a structural feature important for the antifungal activity of PCA [21]. It is known that the PCA completely inhibited in vitro [22] and in vivo [23] the growth of *Seiridium cardinale*, a fungus that induces canker of common Italian cypress (*Cupressus sempervirens* L.). Another example is *Emericella* sp. SMA01, a marine symbiotic fungus that produced PCA as the main metabolite with antifungal activity against *Phytophthora capsici*, *Gibberella zeae*, and *Verticillium dahliae* with IC_50_ values of 23.26–53.89 μg/mL [24]. PCA was also produced by *Streptomyces kebangsaanensis* isolated from the stem of a Malaysian ethnomedicinal plant, *Portulaca oleracea* in 2013. PCA inhibited *Fusarium solani* isolates, UZ541/12, and UZ667/13 at a minimal inhibitory concentration of 18.00 μg/mL [25]. Phenazine derivatives also showed inhibitor activity toward clinical antibiotic-resistant bacteria such as *Mycobacterium tuberculosis* in the range 18.3 to 146.5 μM [26].

This study reports the isolation, chemical characterization, and antifungal activity of phenazine and mesaconic acid produced by *P. fluorescens* 9 against *M. phaseolina*, *C. nicotianae*, and *C. truncatum*, all isolated from soybean in Argentina. The antifungal activity of phenazine was compared to that of PCA and 2-OH P, as well as some other semisynthetic derivatives prepared by nitration of phenazine to evaluate their potential as natural fungicides. The results of a structure–activity relationship (SAR) study are also discussed.

## 2. Materials and Methods

### 2.1. General Experimental Procedures

A Bruker spectrometer (Karlsruhe, Germany) working at 400 MHz was used to record ^1^H-NMR spectra in CDCl_3_ which was used as an internal standard. The LC/MS TOF system Agilent 6230B, HPLC 1260 Infinity was used to record ESI mass spectra. The Phenomenex LUNA (C18 (2) 5 μm 150 × 4.6 mm column was used for HPLC separation. Analytical and preparative thin-layer chromatography (TLC) was performed on silica gel plates (Kieselgel 60, F_254_, 0.25 and 0.5 mm respectively) or on reverse-phase (Whatman, KC18 F_254_, 0.20 mm, plates (Merck, Darmstadt, Germany). UV light and/or iodine vapors were used to visualize the compounds (CC: silica gel, Merck, Kieselgel 60, 0.063–0.200 mm). The samples of standard phenazine and meseconic acid were purchased from Sigma Aldrich. The samples of PCA and 2OH P (**3** and **4**) were obtained from the culture filtrate of *P. chlororaphis* subsp. *aureofaciens* strain M71, as previously reported [21].

### 2.2. Bacterial and Fungal Strains 

*P. fluorescens* 9 and *B. subtilis* 54 strains were isolated from soil samples and healthy soybean plants collected in the field from different locations in the Santa Fe Province, Argentina as previously reported [19]. The *M. phaseolina* 2013-1 strain was obtained from infected soybean roots grown in Pergamino, Buenos Aires, Argentina, as previously reported [13]. *Cercospora nicotianae* isolate Cn_2017_BOL34 was obtained from soybean leaves with Cercospora leaf blight symptoms sampled from commercial soybean fields in Santa Cruz, Bolivia in 2017 as previously reported [15]. The strain 17-5-1 of *Colletotrichum truncatum* was isolated from soybean leaves with anthracnose symptoms sampled from commercial soybean fields in Roldán, Santa Fe, Argentina in 2017. The strain identified was kept in the culture collection of the Department of Plant Pathology, FAUBA.

### 2.3. Cell-Free Supernatants

Bacteria were grown in minimal medium M9 (composition according to the Cold Spring Harb Protoc 2010 for 1 L: (1×) M9 salts mixture from Sigma-Aldrich (Saint-Louis, MO, USA) supplemented with 20% glucose, 1 M MgSO_4_, and 1 M CaCl_2_) at 28 ± 2 °C for 72 h with shaking at 150 rpm. Then, the cells were removed by centrifugation (7000× *g* for 30 min), and supernatants were filtered using 0.22 μm pore diameter membranes (Corning^®^, New York, NY, USA) and concentrated 1:10.

### 2.4. Production, Extraction, and Purification of Metabolites from Pseudomonas Fluorescens 9 and Production of the Crude Extract of Bacillus subtilis 54

*P. fluorescens* 9 and *B. subtilis* 54 were grown separately in minimal medium M9 for 72 h with shaking at 150 rpm. Cells were removed by centrifugation (7000× *g* for 30 min), and supernatants were filtered using 0.22 µm pore diameter membranes (Corning^®^). The culture filtrate (1 L for each bacterium) was lyophilized, and the material obtained was dissolved in distilled water (100 mL). A 50 mL aliquot was alkalinized with ammonia (37%) up to pH 10 and extracted with ethyl acetate (3 × 50 mL). The organic extracts were combined, washed with distilled water, and dehydrated with Na_2_SO_4_. The filtrates were evaporated under reduced pressure, obtaining an amorphous yellow solid residue (15 mg for *P. fluorescens* 9) and white solid residue (8 mg for *B. subtilis* 54). Only the crude extract of *P. fluorescens* 9 was purified by TLC on silica gel eluted with chloroform–*iso*-propanol (97:3) yielding a pure homogenous solid that was identified, as reported below, as phenazine (**1**, 2.1 mg). The other 50 mL of the initial culture filtrate was acidified with formic acid up to pH 2 and extracted with ethyl acetate (3 × 50 mL). The organic extracts were treated as above reported to give a white crystal identified, as reported below, as mesaconic acid (**2**, 3.5 mg).

1. Phenazine (**1**): yellow amorphous solid, ^1^H-NMR δ: 8.29 (4H, dd, *J* = 6.5 and 3.3 Hz), 7.88 (4H, dd, *J* = 6.5 and 3.3 Hz). ESI MS (+) *m*/*z*: 181 [M + H]^+^.
2. Mesaconic acid (**2**): white crystals, ^1^H-NMR (CD_3_OD) δ: 6.76 (1H, q, *J* = 1.4 Hz), 2.23 (3H, d, *J* = 1.4 Hz). ESI MS (−): *m*/*z* 129 [M − H]^−^.

### 2.5. Nitration of Phenazine

Phenazine (**1**, 200 mg) was added to a mixture of concentrated sulfuric acid (0.5 mL) and nitric acid (37%, 0.5 mL). The reaction was carried at 50 °C for 4 days. The solution was poured into ice water, and its pH was adjusted to 9–10 with concentrated NaOH (**12** N), before extraction with ethyl acetate (3 × 70 mL). The organic extracts were combined, dried (Na_2_SO_4_), filtered, and evaporated under reduced pressure, yielding a yellow solid residue (150 mg). This was purified on a silica gel column eluted with methylene chloride. Ten homogeneous fractions were obtained (F1–F10). F2 (3.2 mg) was isolated as a yellow amorphous solid and identified as 1,9-dinitrophenazine (**7**). F3 (10.9 mg) was further purified by TLC eluted with methylene chloride, yielding a yellow amorphous solid (2.5 mg) identified as 2,9-dinitrophenazine (**8**). F5 (28.4 mg) was further purified by TLC eluted with methylene chloride–*iso*-propanol 98:2, yielding a yellow amorphous solid (23.5 mg) identified as 1,3-dinitrophenazine (**6**). Lastly, F8 (14.4 mg) was further purified by TLC eluted with methylene chloride–*iso*-propanol 95:5, yielding a yellow amorphous solid (**5**, 3.0 mg) identified as 2-nitrophenazine.

1. 2-Nitrophenazine (**5**): yellow amorphous solid, ^1^H-NMR δ: 9.24 (1H, d, *J* = 3.4 Hz, H-1), 8.60 (1H, dd, *J* = 9.5 and 3.4 Hz, H-3), 8.43 (1H, d, *J* = 9.5 Hz, H-4), 8.33 (2H, m, H-6 and H-9), 7.99 (2H, m, H-7 and H-8), Figure S1. ESI MS (+) *m*/*z*: 226 [M + H]^+^.
2. 1,3-Dinitrophenazine (**6**): yellow amorphous solid, ^1^H-NMR δ: 9.28 (1H, d, *J* = 2.2 Hz, H-4), 9.26 (1H, d, *J* = 2.2 Hz, H-2), 8.70 (2H, m, H-7 and H-8), 8.51 (2H, m, H-6 and H-9), Figure S2. ESI MS (+) *m*/*z*: 271 [M + H]^+^, ESI MS (−) *m*/*z*: 269 [M − H]^−^.
3. 1,9-Dinitrophenazine (**7**): yellow amorphous solid, ^1^H-NMR δ: 8.60 (2H, br d, *J* = 8.6 Hz, H-4 and H-6), 8.30 (2H, dd, *J* = 7.7 and 1.2 Hz, H-2 and H-8), 8.04 (2H, m, H-3 and H-7), Figure S3. ESI MS (+) *m*/*z*: 271 [M + H]^+^, ESI MS (−) *m*/*z*: 269 [M − H]^−^.
4. 2,9-Dinitrophenazine (**8**): yellow amorphous solid, ^1^H-NMR δ: 9.26 (1H, d, *J* = 2.5 Hz, H-1), 8.69 (1H, dd, *J* = 9.0 and 2.5 Hz, H-3), 8.59 (1H, br d, *J* = 8.9 Hz H-6), 8.53 (1H, d, *J* = 9.0 Hz, H-4), 8.39 (1H, br d, *J* = 8.9 Hz, H-8), 8.04 (1H, t, *J* = 8.9 Hz, H-7), Figure S4. ESI MS (+) *m*/*z*: 271 [M + H]^+^, ESI MS (−) *m*/*z*: 269 [M − H]^−^.

### 2.6. Crystal Structure Determination of Mesaconic Acid (***2***)

Single crystals of mesaconic acid (**2**) suitable for X-ray structure analysis were obtained by slow evaporation of a solution of 2 in ethyl acetate. One selected crystal was mounted in flowing N_2_ at 173 K on a Bruker Nonius Kappa CCD diffractometer equipped with Oxford Cryostream apparatus (graphite monochromated MoKα radiation λ = 0.71073 Å, CCD rotation images, thick slices, φ and ω scans to fill asymmetric unit). The structure was solved by direct methods using the SIR97 program [27] and anisotropically refined by the full matrix least-squares method on F^2^ against all independent measured reflections using the SHELXL-2018/3 program [28]. The hydroxy H atoms were located in difference Fourier maps and freely refined with Uiso (H) equal to 1.2 Ueq of the carrier atom. All the other hydrogen atoms were introduced in calculated positions and refined according to the riding model. Crystals were of poor quality, and the structure was refined as a two-component twin using HKLF5 procedure. Crystallographic data of 2: empirical formula: C_5_H_6_O_4_; formula weight: 130.10 g·mol^−1^; monoclinic, P 2_1_/c; a: 7.079(**2**) Å; b: 11.8200(**7**) Å; c: 6.8680(**15**) Å; β: 97.92(**2**)°; V: 569.2(**2**) Å^3^; Z: 4, Dx: 1.518 Mg/m^3^; independent reflections: 4831; final R indices [I > 2sigma(I)]: R1 = 0.1347, wR2 = 0.3625; largest diffraction peak and hole: 0.665 and −0.697 e.A^−3^. All crystallographic data for 2 were deposited in the Cambridge Crystallographic Data Center with deposition number CCDC 2121226. These data can be obtained free of charge from www.ccdc.cam.ac.uk/data_request/cif (accessed on 19 November 2021). The room-temperature crystal structure of mesaconic acid was previously reported by Gupta et al. [29].

### 2.7. Antifungal Assay

To test the cell-free supernatants (CSFs) of *P. fluorescens* 9 and *B. subtilis* 54, 100 μL aliquots were placed on the potato dextrose agar (PDA) plate at 1.5 cm from the fungal disc (6 × 6 mm diameter) of *M. phaseolina*. As a positive control, fungicidal pentachloronitrobenzene ≥94% (PCNB) (Sigma-Aldrich, Saint-Louis, MO, USA) dissolved in toluene was used. Toluene alone was used as a negative control. The experiment was performed in triplicate; the plates were incubated at 28 °C for 5 days and examined for zones of inhibition of grown colonies [30].

The crude extracts of both bacteria were dissolved in 4% methanol and tested against *M. phaseolina* at a final concentration of 10 mg/mL as described above. To detect the antifungal activity of compounds obtained by *P. fluorescens* 9 phenazine (**1**), of its natural analogs (**3** and **4**), and of semisynthetic derivatives (**5**–**8**), as well as that of mesaconic acid (**2**), against *M. phaseolina*, *C. nicotianae*, and *C. truncatum*, the dual-culture plate method was carried out as previously described by Puopolo et al. [21] with some modifications. The fungi were grown in Potato Dextrose Agar (PDA) separately at 28 ± 2 °C for 5–7 days. Fungal plugs of 6 × 6 mm diameter were placed at the center of PDA plates, and each compound at 25, 50, 100, 200, 300, 500, and 1000 μg/mL, with a final concentration of 4% methanol, was placed on the opposite four sides of the plates 1.5 cm away from the fungal disc. Plates containing the fungal plugs alone were used as a positive control. As a negative control, 4% MeOH was applied on the top of each fungal plug positioned in the center of the Petri dish. All plates were incubated at 28 ± 2 °C for 5–7 days, and the experiments were performed in triplicate. The percentage inhibition of fungal growth was calculated using the following formula:

% = [(Rc − Ri)/Rc] × 100(1)

where Rc is the radial growth of the test fungi in the control plates (mm), and Ri is the radial growth of the fungi in the presence of different compounds tested (mm). The results show the antifungal activity of different compounds analyzed by ANOVA using Tukey’s test.

### 2.8. Minimal Inhibitory Concentration (MIC_50_)

The minimal inhibitory concentration (MIC_50_) was determined using the broth microdilution method in 24-well microplates, as described by Mefteh et al. [31] with some modifications. Serial dilution of each compound (**3**, **1**, and **5**–**8**) was prepared to get final concentrations ranging from 1 to 100 μg/mL dissolved in 4 % of MeOH. Each well contained ultrapure Milli-Q water with different tested compounds dissolved and a fungal plug (4 × 4 mm) resuspended in 2× PD broth in a final volume of 500 μL. The wells containing the different fungal plugs with only PD broth were used as a positive control and with the added of 4% MeOH as a negative control. The experiment was performed in triplicate. The plates were incubated at 28 °C for 7 days. The MFC values of different tested compounds (**1**, **3**, and **5**–**8**) were interpreted as the concentrations able to inhibit 50% of fungal growth. Finally, the percentage inhibition of fungal growth was calculated using the formula described above.

### 2.9. Data Analysis

All statistical analyses were performed using GraphPad Prism (GraphPad 243 Software, San Diego, CA), and the data were expressed as the mean ± SEM. Differences among groups were compared by ANOVA or *t*-test as indicated in figure legends. Differences were considered statistically significant at *p* < 0.05.

## 3. Results and Discussion

**Figure 1:**
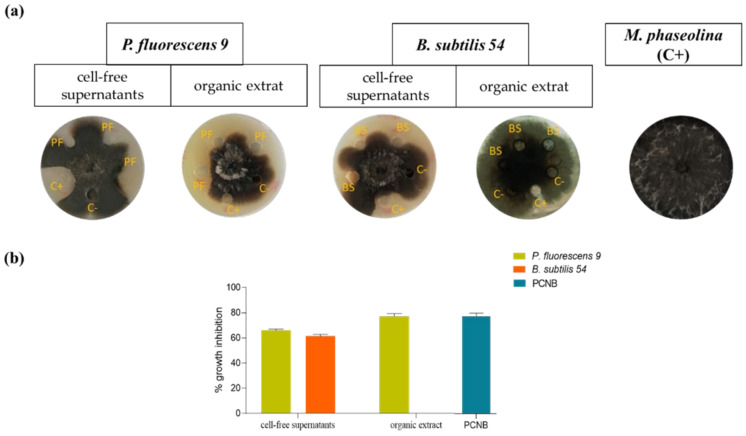
Antifungal activity of cell-free supernatants (CSFs) and respective organic extracts of *P. fluorescens* 9 and *B. subtilis* 54 against *M. phaseolina*. (**a**) CSFs of bacterial strains collected after 72 h of growth and the respective organic extracts were tested for their ability to inhibit the mycelial growth of *M. phaseolina*. C+: positive control, pentachloronitrobenzene (PNCB) 0.5 mg/mL; C−: negative control, toluene. All experiments were performed in triplicate with three independent trials. (**b**) The percentage inhibition of fungal growth was reported as the percentage reduction in the diameter of the fungal mycelia compared to the control plate. Data are presented as means ± standard deviation (*n* = 3). For comparative analysis of groups of data, one-way ANOVA was used; *p*-values were extremely significant (*p* < 0.0001).

Both the culture filtrates and their corresponding organic extracts obtained from the selected strains *P. fluorescens* 9 and *B. subtilis* 54 [19] were tested for their ability to inhibit the growth of *M. phaseolina* in vitro. As shown in Figure 1, the CSFs of *P. fluorescens* 9 and *B. subtilis* 54 inhibited the fungus growth by 65% and 60%, respectively, in agreement with the results previously reported [19]. However, when the corresponding crude organic extracts were tested, only that of the first bacterium confirmed its strong antifungal activity, inhibiting the growth of *M. phaseolina* by about 75% (Figure 1).

**Figure 2:**
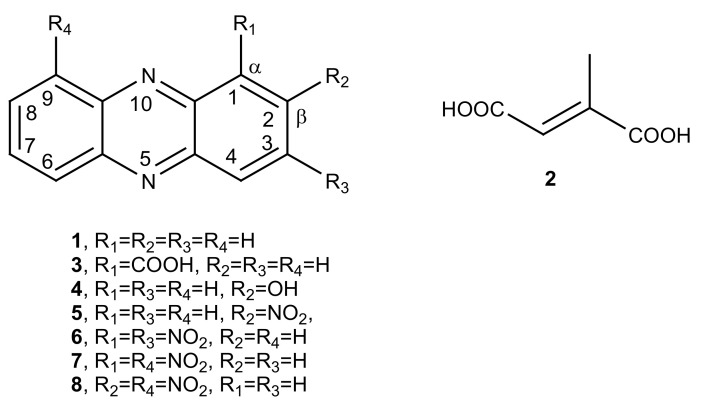
Structure of phenazine (**1**), its natural analogs 1-carboxy and 2-hydroxy-phenazine (**3** and **4**), and four mono- and dinitro derivatives (**5**–**8**) and mesaconic acid (**2**).

**Figure 3:**
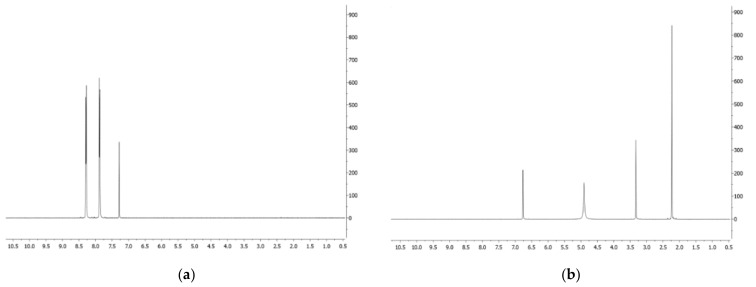
(**a**) ^1^H-NMR spectrum of phenazine (**1**) recorded in CDCl_3_ at 400 MHz; (**b**) ^1^H-NMR spectrum of mesaconic acid (**2**) recorded in CD_3_OD at 400 MHz.

**Figure 4:**
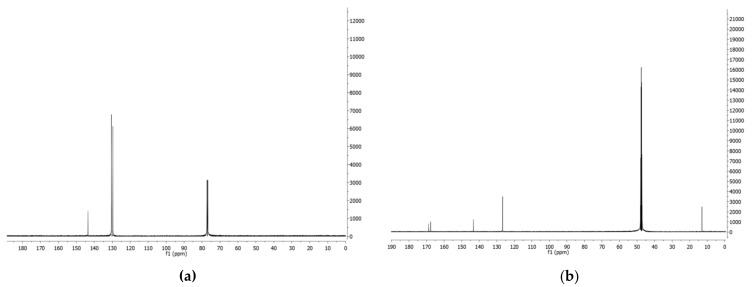
(**a**) ^13^C-NMR spectrum of phenazine (**1**) recorded in CDCl_3_ at 125 MHz; (**b**) ^13^C-NMR spectrum of mesaconic acid (**2**) recorded in CD_3_OD at 125 MHz.

Thus, the organic extract of *P. fluorescens* 9 culture filtrates was fractionated, as reported in detail Section 2, to yield phenazine and mesaconic acid (2-methyl-1,4-butenoic acid), as indicated by numbers **1** and **2**, respectively, in Figure 2. Both **1** and **2** were identified by ^1^H-NMR (Figure 3a,b) and ^13^C-NMR (Figure 4a,b), as well as ESI MS spectra, in comparison with those of commercially available standards recorded in the same conditions. The standards were also used in co-chromatography analyses developed in different solvent mixtures. Mesaconic acid was crystallized by slow evaporation of ethyl acetate solution, and X-ray analysis was carried out. The crystal data corresponding to that structure have already published [29]. 

**Figure 5:**
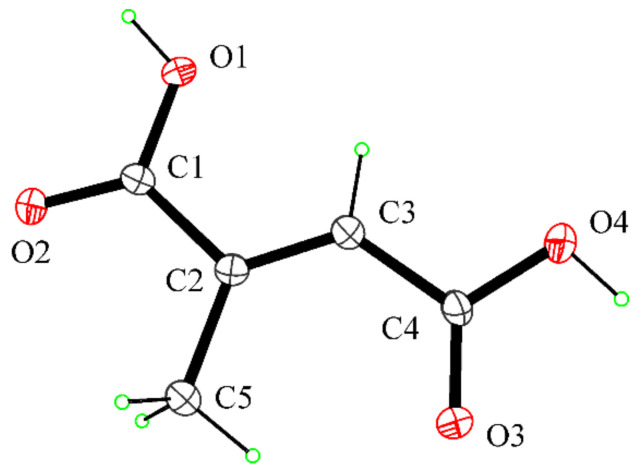
ORTEP view of the molecular structure of **2**. Thermal ellipsoids were drawn at the 30% probability level.

Mesaconic acid (**2**) was crystallized by slow evaporation of ethyl acetate solution. The compound was undoubtedly identified by the X-ray analysis as mesaconic acid, and the molecular structure is reported in Figure 5. The crystal structure of mesaconic acid, previously reported at room temperature by Gupta et al. [29], was redetermined at 173 K. 

Mesaconic acid and several other organic acids having a methyl or methylene side-chain have been reported, as well as their interconversion biological systems. Mesaconate hydration is also the key biosynthetic step to obtain d-citromalate in *P. fluorescens* [32]. The natural occurrence of mesaconic acid in a variety of plants and animals has also been reported [33].

Phenazines have primarily been isolated from *Pseudomonas*, *Streptomyces*, as well as from a few other soil or marine organisms. They show several biological activities including antimalaria, antibiotic, antitumor, and antiparasitic activities, which were reviewed together with their biosynthesis and the preparation of semisynthetic derivatives [34]. More than 100 different natural phenazine analogs and over 6000 synthetic compounds have been investigated as potential anticancer agents, and the results were critically reviewed [35].

The antifungal activity of phenazine and its natural analogs is of particular interest for their potential application in agriculture as biofungicides. In fact, the isolation of PCA (**3**) (Figure 2) from *P. chlororaphis* subsp. *aureofaciens* strain M7, as well as its ability to completely inhibit *S. cardinale* in vitro [22] and in vivo [23], was previously mentioned. 1-Hydroxyphenazine together with cereusitin was also produced, as the main metabolite, from a strain *P. aeruginosa* 2016NX1, isolated from the root of *Millettia specisoa*. 1-Hydroxyphenazine strongly inhibited the growth of several common plant-pathogenic fungi and bacteria such as *Cochliobolus miyabeanus*, *Diaporthe citri*, *Salmonella* sp., and *Klebsiella oxytoca,* and its potential as a biocontrol agent was evaluated [36]. Instead, 2-hydrophenazine (**4**) as reported above exhibited only slight antifungal activity. *P.* *aeruginosa* KU_BIO2, obtained from soil, was also able to produce a pyocyanin blue-green phenazine pigment which showed interesting ecofriendly medicine, agriculture, and environment applications. In fact, it showed remarkable dye properties and significant inhibition of *Magnaporthe grisea* and *Xanthomonas oryzae* growth, causal agents of two different severe rice diseases [37]. A mutation in the gltA gene of the *P. chlororaphis* subgroup from a native isolate from Argentina was recently reported as a determinant inductor of a phenotypic change associated with phenazine production, which is essential for the bacterial antifungal activity [38]. In addition, phenazine derivatives were also produced by fluorescent pseudomonads (FPs) proposed for the control of sheath blight of rice [39].

**Figure 6:**
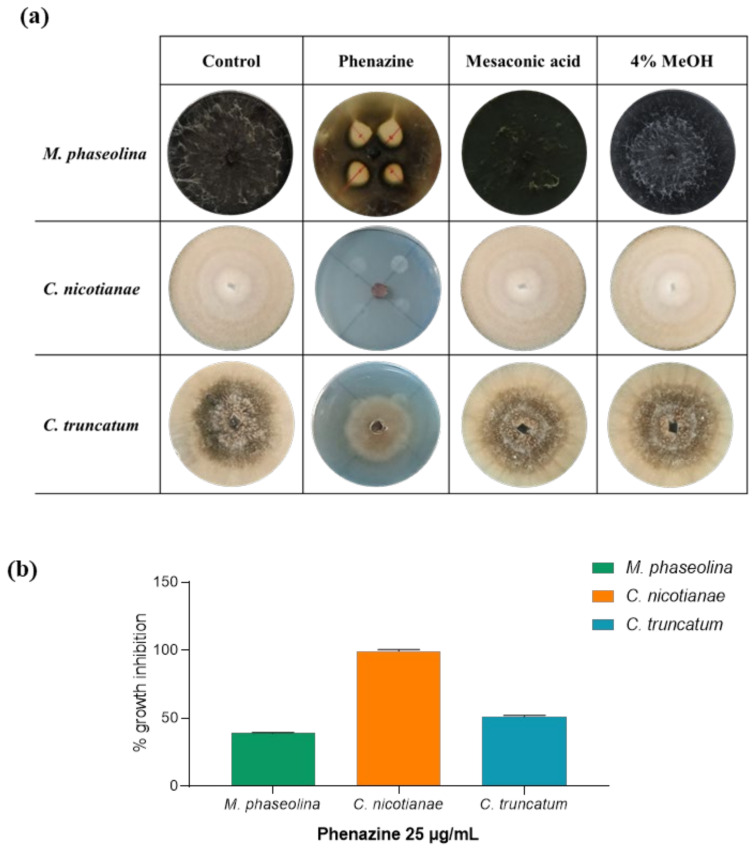
Effects of phenazine (**1**) and mesaconic acid (**2**) produced by *P. fluorescens* 9. The graphic shows the ability of phenazine to inhibit the fungal growth at a concentration of 25 µg/mL. (**a**) Representative photos of the antifungal assay for in vitro inhibition of mycelial growth of *M. phaseolina*, *C. nicotianae*, and *C. truncatum:* positive control (fungi alone), phenazine, mesaconic acid, and negative control (4% MeOH). (**b**) Inhibition of fungal growth by phenazine reported as the percentage reduction in the diameter of the fungal mycelia in the treated plate compared to that in the control plate. Data are presented as means ± standard deviation (*n = 3*) compared to control fungi grown alone. For comparative analysis of groups of data, one-way ANOVA was used; *p*-values were extremely significant (*p* < 0.001).

Thus, phenazine and mesaconic acid (**1** and **2**) isolated from *P. fluorescens* 9 (which are FPs) were spot-inoculated in PDA plates to test for their antifungal activity against some phytopathogenic fungi isolated from infected soybean, *M. phaseolina*, *C. nicotianae*, and *C. truncatum,* as described in Section 2. As shown in Figure 6, only phenazine exhibited a strong antifungal activity when spot-inoculated (25 µg/mL), inhibiting the growth of *M. phaseolina* by around 40%, *C. nicotianae* by 100%, and *C. truncatum* by around 50%. Mesaconic acid tested up to 1 mg/mL used in the spot inoculation against the same pathogenic fungi did not show any antifungal activity. The variation in activity (75% against 40%) observed when testing the crude extract (Figure 1) and phenazine (Figure 6) on *M. phaseolina* is probably due to the other metabolites present in the crude organic extract obtained from CSFs of *P. fluorescens* 9. These compounds, although present in very low amounts, as shown by TLC analysis, could probably act synergically with phenazine, obtaining major inhibitory activity.

Considering these results, the activity of phenazine (**1**) was compared with that of PCA (**3**), 2-OH P (**4**) (for this analog, only against *M. phaseolina*), and some synthetic derivatives, which were prepared in order to study the effect of one or more electron attractor groups such as NO_2_ linked to the phenazine carbon skeleton at the two different positions α (C-1,C-4, C-6, and C-9) and β (C-2, C-3, C7, and C-8). 

In fact, nitration of phenazine, carried out with a classic sulfur–nitric acid mixture [40] at room temperature for a long time (4 days), yielded 2-nitro, 1,3-dinitro, 1,9-dinitro, and 2,9-dinitro phenazine (**5**–**8**). These derivatives were characterized by their ^1^H-NMR and ESI MS spectra (as detailed in Section 2).

**Figure 7:**
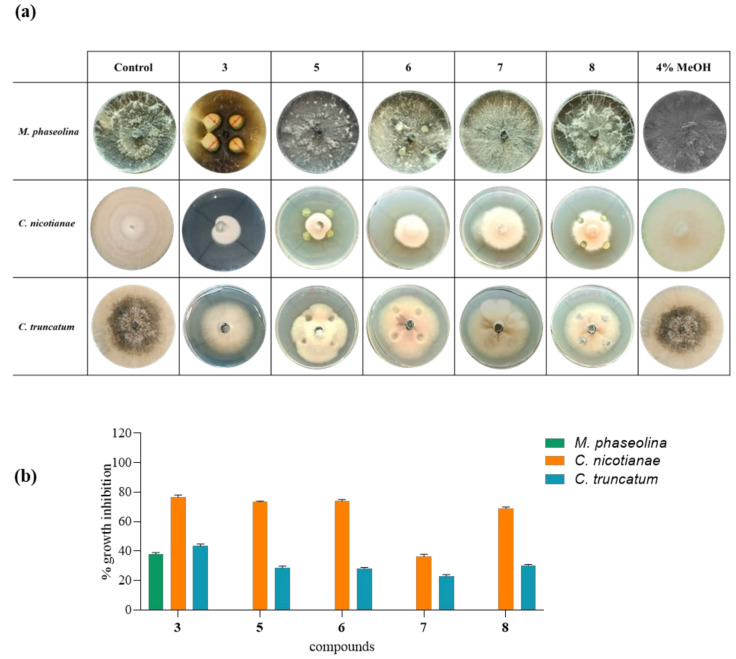
Effects of PCA (**3**) and phenazine derivates (**5**–**8**) against *M. phaseolina, C. nicotianae*, and *C. truncatum*. The graphic shows the fungal inhibition growth by tested compounds at a concentration of 25 µg/mL. (**a**) Representative photos of the biological assay for in vitro inhibition of mycelial growth of *M. phaseolina*, *C. nicotianae*, and *C. truncatum*. The positive controls were fungi grown on PDA plates, and the negative control was 4% MeOH. (**b**) Inhibition of fungal growth reported as the percentage reduction in the diameter of the fungal mycelia in the treated plate compared to that in the control plate. **3** = phenazine-1carboxylic acid (PCA); **5** = 2-nitrophenazine; **6** = 1,3-nitrophenazine; **7** = 1,9-dinitrophenazine; **8** = 2,9-dinitrophenazine. Data are presented as means ± standard deviation (*n* = 3) compared to control fungi grown alone. For comparative analysis of groups of data, one-way ANOVA was used; *p*-values were extremely significant (*p* < 0.0001).

The nitrophenazine derivatives (**5**–**8**), compared with phenazine and PCA, were assayed against the pathogenic fungi *M. phaseolina*, *C. nicotianae*, and *C. truncatum* (Figure 5). As shown in Figure 7, PCA (**3**) exhibited antifungal activity when spot-inoculated at 25 µg/mL (the same concentration used for phenazine). This represents the minimal concentration able to inhibit the fungal growth. PCA (**3**) and phenazine exhibited the same percentage of growth inhibition of *M. phaseolina* and *C. truncatum*. In contrast, when tested against *C. nicotianae* PCA (**3**) showed a lower inhibition percentage (78%) than phenazine (100%). When 2-hydroxyphenazine (**4**) was tested against *M. phaseolina* at a final concentration from 25 µg/mL to 1 mg in 10 μL of spot inoculation, it showed no antifungal activity (data not shown).

Finally, all inactive nitrophenazine derivatives (**5**–**8**) were tested against *M. phaseolina* with spot-inoculation in PDA plates at concentrations from 25 µg/mL to 1 mg/mL of spot inoculation, but they did not show antifungal activity. In contrast, all derivates showed antifungal activity against *C. nicotianae* and *C. truncatum*. In particular, the best activity was found against *C. nicotianae* with about 70% inhibition at a final concentration of 25 µg/mL. 

In order to identify the minimal inhibitory concentration able to inhibit the fungal growth of 50%, the tested compounds were subjected to a microdilution plate assay against *M. phaseolina*, *C. nicotianae*, and *C. truncatum*. As shown in Table 1, for compounds **3** and **1**, 35 µg/mL was necessary to inhibit *M. phaseolina* by 50%, 15 μg/mL was necessary to inhibit *C. nicotianane*, and 25–30 µg/mL was necessary to inhibit *C. truncatum*. Regarding the phenazine derivatives, when tested on *C. nicotianae,* compounds **5**, **6**, and **8** showed an MIC_50_ of 20 μg/mL, while that of compound **7** was 40 μg/mL. Instead, all phenazine derivates (**5**–**8**) inhibited *C. truncatum* by 50% at 60 µg/mL.

Phenazine and PCA could be proposed as natural antagonists for the control of not only *M. phaseolina* but also *C. nicotianae* and *C. truncatum*. According to the SAR study and considering the inhibition of *M. phaseolina*, the nature of the carboxylic and 2-hydroxy groups, which are electron attractor and electron repulsor groups, respectively, present in PCA and 2-OH P (**3** and **4**), significantly affects the inhibition of *M. phaseolina*, as **3** had the same strong inhibition as **1**, while **4** was inactive. Instead, the strong inhibition activity of 1-hydroxyphenazine reported on different pathogenic fungi and bacteria [32] indicates that only the position α or β to which the substituents are bonded play a role in imparting activity. Thus, 2-nitrophezine and 1,3-dinitro and 2,9 dinitro-phenazine should be inactive as at least one of the substituents is in the β-position. Lastly, 1,9-dinitro phenazine is probably inactive due to the well-known steric hindrance in this kind of phenazine derivative. Derivatives **5**–**8** affected the other two fungi differently from *M. phaseolina*. In particular, on *C. nicotianae*, all four derivatives **5**–**8** showed a slightly reduced activity with respect to **1** and **3**, whereas, on *C. truncatum*, the inhibition effect of these derivatives was significantly reduced; in particular, derivative **7** showed a markedly reduced inhibition of both fungi. Thus, the activity is probably also dependent on the sensitivity of fungal species.

Future studies will focus on the characterization of these compounds in order to test them at different temperatures and pH, as well as in other environmental conditions, to observe their resistance via in vitro and in vivo experiments in plants infected by phytopathogens. Other studies will focus on optimizing their large-scale production and finding the best formulation for their application in the field.

## Figures and Tables

**Table 1:** The MIC_50_ of PCA (**3**), phenazine (**1**), and phenazine derivates (**5**–**8**) against *M. phaseolina*, *C. nicotianae*, and *C. truncatum*.

MIC_50_ (µg/mL)			
Compounds	*M. phaseolina*	*C. nicotianae*	*C. truncatum*
**3**	35 µg/mL	15 µg/mL	25 µg/mL
**1**	35 µg/mL	15 µg/mL	30 µg/mL
**5**	-	20 µg/mL	60 µg/mL
**6**	-	20 µg/mL	60 µg/mL
**7**	-	40 µg/mL	60 µg/mL
**8**	-	20 µg/mL	60 µg/mL

## Data Availability

Not applicable.

## Supplementary Materials

The following are available online at https://www.mdpi.com/article/10.3390/biom11111728/s1: Figure S1: ^1^H-NMR spectrum of 2-nitrophenazine (**5**), recorded in CDCl_3_ at 400 MHz; Figure S2: ^1^H-NMR spectrum of 1,3-dinitrophenazine (**6**), recorded in CDCl_3_ at 400 MHz; Figure S3: ^1^H-NMR spectrum of 1,9-dinitrophenazine (**7**), recorded in CDCl_3_ at 400 MHz; Figure S4: ^1^H-NMR spectrum of 2,9-dinitrophenazine (**8**), recorded in CDCl_3_ at 400 MHz.
