# Supplementary material for: Pseudomonas fluorescens Showing Antifungal Activity against Macrophomina phaseolina, a Severe Pathogenic Fungus of Soybean, Produces Phenazine as the Main Active Metabolite
Source: Biomolecules. 2021 Nov 19;11(11):1728. doi: 10.3390/biom11111728 (PMC8615785; doi:10.3390/biom11111728)
Supplement: Supplementary file 1 [file biomolecules-11-01728-s001.zip › biomolecules-1457566-supplementary.pdf]

Supporting Information List

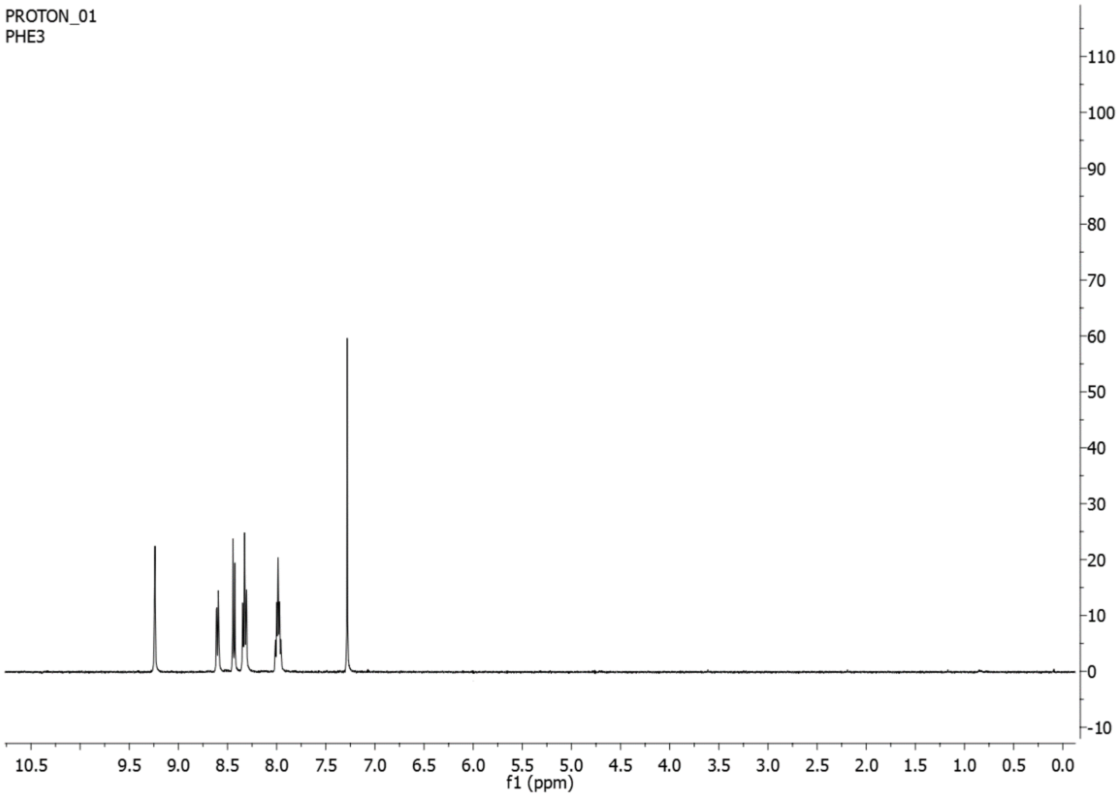

Figure S1. ^{1}H NMR spectrum of 2-nitrophenazine (**5**), recorded in $CDCl_3$ at 400 MHz.

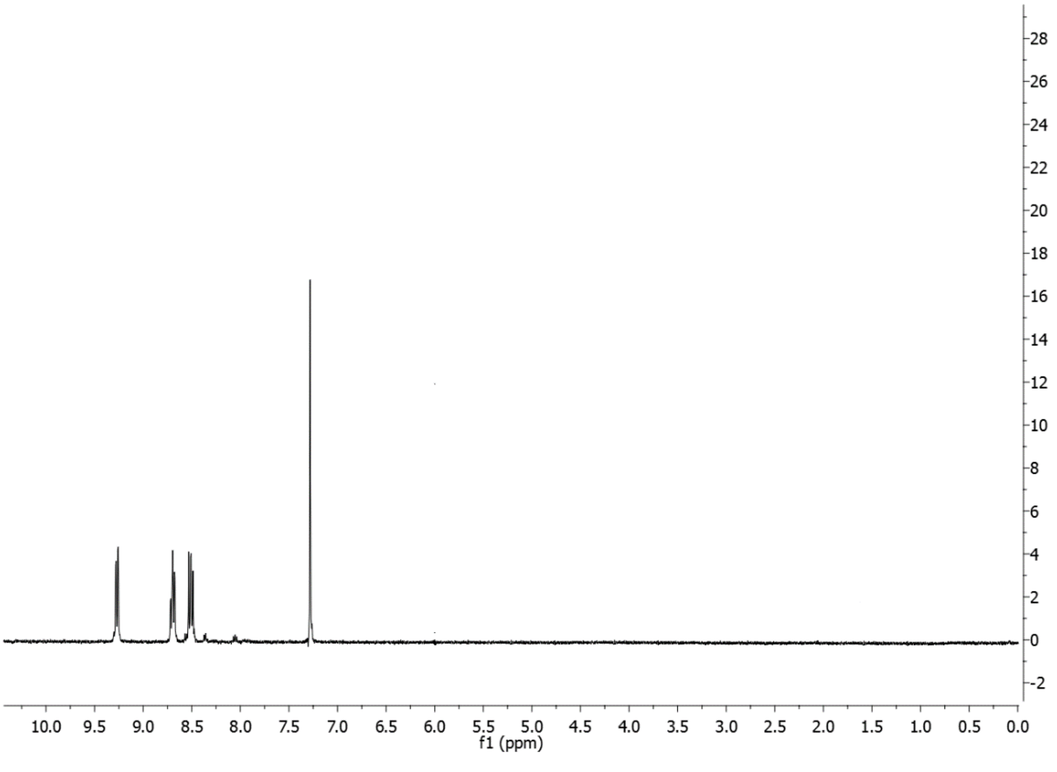

Figure S2. ^{1}H NMR spectrum of 1,3-dinitrophenazine (**6**), recorded in $CDCl_3$ at 400 MHz.

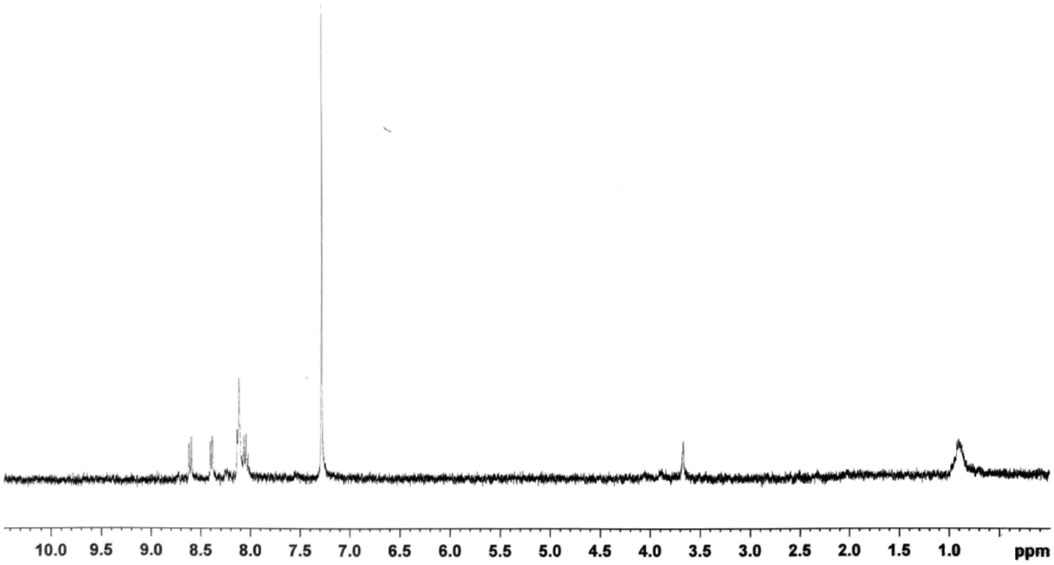

Figure S3. ^{1}H NMR spectrum of 1,9-dinitrophenazine (**7**), recorded in $CDCl_3$ at 400 MHz.

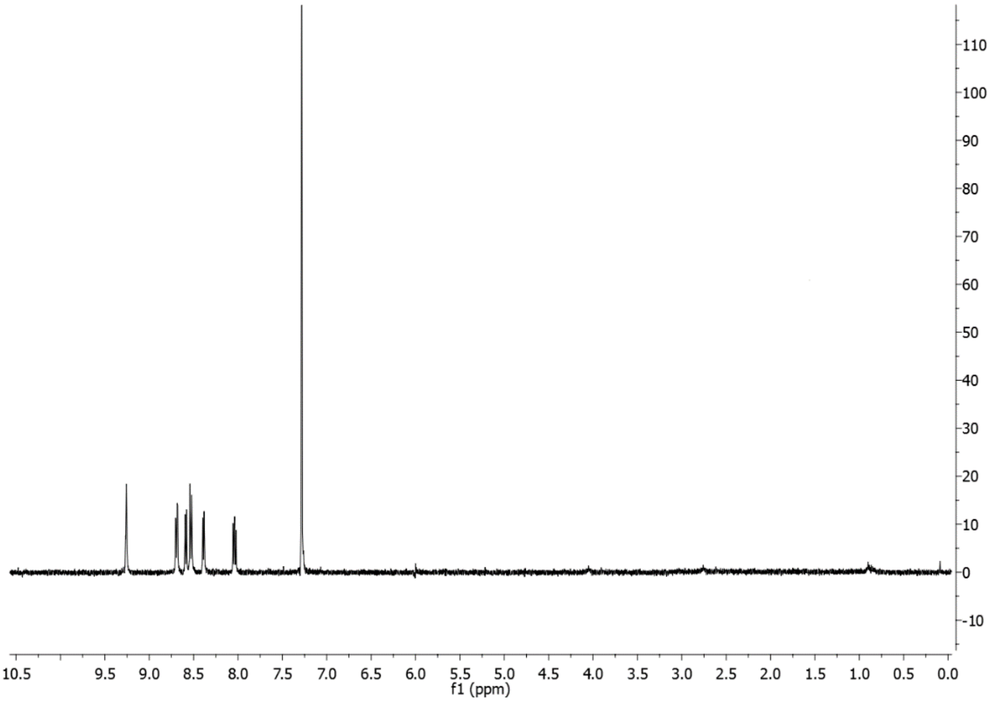

Figure S4. ^{1}H NMR spectrum of 2,9-dinitrophenazine (**8**), recorded in $CDCl_3$ at 400 MHz.
